# Digital karyotyping reveals probable target genes at 7q21.3 locus in hepatocellular carcinoma

**DOI:** 10.1186/1755-8794-4-60

**Published:** 2011-07-19

**Authors:** Hui Dong, Hongyi Zhang, Jianping Liang, Huadong Yan, Yangyi Chen, Yan Shen, Yalin Kong, Shengyue Wang, Guoping Zhao, Weirong Jin

**Affiliations:** 1 Chinese National Human Genome Center at Shanghai, Shanghai 201203, China; 2 Department of Hepatobiliary Surgery, General Hospital of Air Force PLA, Beijing 100036, China; 3 Hunan Province Tumor Hospital, Changsha 410013, China; 4 Ningbo No.2 Hospital, Ningbo 315010, China; 5 National Engineering Center for Biochip at Shanghai, Shanghai 201203, China

## Abstract

**Background:**

Hepatocellular carcinoma (HCC) is a worldwide malignant liver tumor with high incidence in China. Subchromosomal amplifications and deletions accounted for major genomic alterations occurred in HCC. Digital karyotyping was an effective method for analyzing genome-wide chromosomal aberrations at high resolution.

**Methods:**

A digital karyotyping library of HCC was constructed and 454 Genome Sequencer FLX System (Roche) was applied in large scale sequencing of the library. Digital Karyotyping Data Viewer software was used to analyze genomic amplifications and deletions. Genomic amplifications of genes detected by digital karyotyping were examined by real-time quantitative PCR. The mRNA expression level of these genes in tumorous and paired nontumorous tissues was also detected by real-time quantitative RT-PCR.

**Results:**

A total of 821,252 genomic tags were obtained from the digital karyotyping library of HCC, with 529,162 tags (64%) mapped to unique loci of human genome. Multiple subchromosomal amplifications and deletions were detected through analyzing the digital karyotyping data, among which the amplification of 7q21.3 drew our special attention. Validation of genes harbored within amplicons at 7q21.3 locus revealed that genomic amplification of SGCE, PEG10, DYNC1I1 and SLC25A13 occurred in 11 (21%), 11 (21%), 11 (21%) and 23 (44%) of the 52 HCC samples respectively. Furthermore, the mRNA expression level of SGCE, PEG10 and DYNC1I1 were significantly up-regulated in tumorous liver tissues compared with corresponding nontumorous counterparts.

**Conclusions:**

Our results indicated that subchromosomal region of 7q21.3 was amplified in HCC, and SGCE, PEG10 and DYNC1I1 were probable protooncogenes located within the 7q21.3 locus.

## Background

Hepatocellular carcinoma (HCC) is one of the major human malignant tumors worldwide. Accumulation of genetic and epigenetic changes plays an important role in the process of hepatocarcinogenesis. Chromosomal aberration is among the most prevalent genetic alterations observed in HCC [1]. Recurrent chromosomal gains of 1q, 6p, 8q, 17q, and 20q, and losses of 1p, 4q, 5q, 6q, 8p, 9p, 10q, 13q, 16q, 17p, 19p, and 22q in HCC have been documented in previous studies [2-7]. Exploring cancer genome in great details always led to the identification of cancer-associated genes (also referred to as *target genes*) located within the amplified or deleted chromosomal regions. For example, deletion at 1p36 locus in HCC was reported to be one of the common mechanisms for inactivation of RUNX3, a putative tumor suppressor gene located within this region [8]. Elevated expression level of PTK2 and EIF3S3 genes in HCC was demonstrated to be significantly correlated with amplification of 8q23-q24, indicating a potential role for these genes in progression of HCC [9].

Recent innovations on molecular genetic techniques allowed investigators to perform genome-wide analysis of chromosomal aberrations at high resolution. Among these techniques, array-based comparative genomic hybridization (aCGH), high-density single nucleotide polymorphism (SNP) arrays and digital karyotyping have been proved to be the most effective tools in discovering abnormal chromosomal features [7,10,11]. Instead of generating analog signal intensity in the array-based methods, digital karyotyping is characterized by providing direct, unbiased, precise and quantitative measurement of chromosomal aberrations, with a theoretical resolution as high as 0.004 Mb [12].

In the current study, digital karyotyping was applied to detect chromosomal aberrations in HCC. We found that the amplification at 7q21.3 locus may contribute to the development or progression of HCC. Gains of 7q21 have been observed in tumors such as prostate cancer, colorectal cancer and HCC [13-15]. Tsuji *et al*. has identified PEG10 as a putative oncogene located within the amplified 7q21 locus in HCC [16]. Our study highlighted that more target genes including SGCE, DYNC1I1, SLC25A13, as well as PEG10, were harbored within the amplicons at 7q21.3 in HCC. Furthermore, up-regulation of SGCE, DYNC1I1 and PEG10 genes were also observed in HCC. These results indicated that amplification of 7q21.3 may be involved in carcinogenesis of HCC, and SGCE, DYNC1I1 and PEG10 were candidate oncogenes located within 7q21.3 locus.

## Methods

### Tissue samples

Clinical tumorous and corresponding non-tumorous liver tissues were obtained from HBV-positive primary HCC patients who underwent curative surgery. Specimens were kept frozen in liquid nitrogen immediately to avoid any degradation of RNA and/or DNA. Blood of normal individuals was obtained from healthy people who underwent regular physical examination. All samples were collected with informed consent and the study was approved by the Institutional Human Subjects Protection Review Board.

### Digital karyotyping

Genomic DNA was isolated from one tumorous liver tissue using DNeasy kit (Qiagen) according to the manufacture's instruction. Then, 10 μg of genomic DNA was used to generate digital karyotyping library as described by Leary et al [17]. However, the concatemers of ditags were not cloned into pZero because 454 Genome Sequencer FLX System (Roche) was applied in large scale sequencing in this study. Instead, the concatemers with adhesive ends of CATG were ligated to a pair of modified adaptors A^+ ^and B^+ ^which provide priming regions for the following emulsion PCR and 454 pyrosequencing. The original Adaptor A and B provided by the GS FLX Standard DNA Library Preparation Kit (Roche) were blunt-ended and could not be ligated to concatemers directly. Thus, we designed a modified Adaptor A^+ ^and B^+ ^with an extruding end which was complementary to the adhesive end of concatemers. The sequences of Adaptor A^+ ^and B^+ ^were as followings: Adaptor A^+ ^forward strand: 5'-CCA TCT CAT CCC TGC GTG TCC CAT CTG TTC CCT CCC TGT CTC AGA CTG CAT G-3'; Adaptor A^+ ^reverse strand: 5'-CAG TCT GAG ACA GGG AGG GAA CAG ATG GGA CAC GCA GGG ATG AG-3'; Adaptor B^+ ^forward strand: 5'-Biotin-CCT ATC CCC TGT GTG CCT TGC CTA TCC CCT GTT GCG TGT CTC AGA CTG CAT G-3'; Adaptor B^+ ^reverse strand: 5'-CAG TCT GAG ACA CGC AAC AGG GGA TAG GCA AGG CAC ACA GGG GA-3'. The underlined basepairs were optional barcode sequences. The following procedures were carried out according to the original protocol [18], and sequencing run was performed on 454 GS FLX instrument. The 21 bp genomic tags were extracted using SAGE2000 software (Invitrogen). The experimentally observed tags were mapped to virtual tags on the genome and their relative frequency was counted to detect genomic amplifications and deletions using Digital Karyotyping Data Viewer software http://cgap.nci.nih.gov/SAGE/DKViewHome. The database of virtual genomic tags was established according to sequences of NCBI Human Genome Assembly Build 37 (released on Aug 2009).

### Quantitative PCR

Genomic DNAs were extracted from tumorous liver tissues of 52 HCC patients and peripheral blood lymphocytes of 52 normal individuals using DNeasy kit (Qiagen). PCR reaction mixture of 25 μl was composed of 50 ng genomic DNA, 12.5 μl SYBR Premix Ex Taq (TaKaRa), 250 nM of each primer and appropriate volume of ddH_2_O. The thermal cycles were performed on ABI Prism 7300 apparatus (Applied Biosystems) with conditions of 95°C for 10 s, followed by 40 cycles of 95°C for 5 s and 60°C for 30 s. Each PCR reaction was performed in triplicate and threshold cycle numbers were averaged. The primers (Table 1) were designed using Primer 3 software http://frodo.wi.mit.edu/primer3/. The repetitive element LINE-1, which is likely to has an equivalent copy number in cancer and normal genomes, was used as an endogenous control for normalization of DNA content.

**Table 1:** Primers used for real-time quantitative PCR

Genes	Primers	Product Size
LINE-1	F: AAAGCCGCTCAACTACATGG R: TGCTTTGAATGCGTCCCAGAG	149 bp
SGCE	F: CAGAGACCTTGCTTCCCTTG R: CGTCTTATCAGCCACTGCAA	193 bp
DYNC1IC	F: ATACAGGACACCCAGGCAAG R: ACTTCTCGGACCATGTCAGG	138 bp
SLC25A13	F: AGGCAGAGCTGGCAAAATAA R: TCATGCCTTTTTGATGTGGA	105 bp
PEG10	F: CCAGTTGTCATCCACCACAG R: TGCAATTTTCCACAGACCAA	219 bp

### Quantitative reverse transcription-PCR (RT-PCR)

Total RNAs were extracted from 32 pairs of tumorous and corresponding non-tumorous liver tissues using TRIzol reagent (Invitrogen), and then treated with RNase-free DNaseI (Promega) prior to reverse transcription. The reverse transcription reactions and following quantitative PCR reactions, as well as statistical analysis were done as previously described [19]. GAPDH was used as internal control for normalizing mRNA level. The sequences of primers used for quantitative RT-PCR were listed in Table 2.

**Table 2:** Primers used for real-time quantitative RT-PCR

Genes	Primers	Product Size
GAPDH	F: ATGGGTGTGAACCATGAGAAG R: AGTTGTCATGGATGACCTTGG	106 bp
SGCE	F: ATGCAAACACCAGACATCCA R: TCTGATGTGGCAAGTTCTGC	220 bp
DYNC1IC	F: TGTGGTCCCCCGTGCATCCT R: AGGGCGGATGCCCCCTCAAT	127 bp
SLC25A13	F: TGGCTGAGGCCCAGAGGCAG R: GCTCCAGCAACAGAACCCAGACC	106 bp
PEG10	F: TCCTGTCTTCGCAGAGGAGT R: TTCACTTCTGTGGGGATGGA	111 bp

### Statistical analysis

Student's *t *test and Bonferroni correction were applied in the statistical analysis of quantitative PCR and RT-PCR data.

## Results

### Digital karyotyping of HCC

We applied 454 pyrosequencing in the current study, and obtained a total of 821,252 genomic tags from the digital karyotyping library of HCC, among which 529,162 tags could be mapped to unique loci of human genome (i.e. filtered tags). By aligning these filtered tags across each chromosome using Digital Karyotyping Data Viewer software with a window size of 20, we identified multiple subchromosomal regions of amplification (fold change ≥3) and deletion (fold change ≤0.1) (Additional file 1). Among all these chromosomal aberrations, gains of chromosome 7 were of most interest. As many as fifty amplicons with sizes ranging from 287 bp to 212,814 bp were detected on the q arm of chromosome 7, some of which harbored known genes while some did not (Table 3). The amplified subchromosomal region between 116,320,004 bp and 116,359,984 bp of 7q31 harbored MET gene, a well-known oncogene involved in hepatocarcinogenesis [20,21]. The SGCE gene previously reported to be up-regulated in HCC by our group [19], was identified to reside within an amplicon at 94,187,248 bp--94,227,832 bp of 7q21.3. According to the power calculation method developed by Salani *et al*, a 3-fold amplification covering 38 kb could be detected with a positive predictive value (PPV) ≥95% if 890,628 experimental tags were obtained [22]. Thus, with 821,252 tags available in this study, the PPV of detecting a 3-fold amplification of 40,584 bp which spans SGCE gene was supposed to be approximately 95%. We also observed that another two genes, DYNC1I1 and SLC25A13 located closely to SGCE, were contained within the amplicons at 7q21.3. Gene expression profile analysis of HCC has implied that SGCE and PEG10 harbored within 7q21.3 locus were putative tumor-associated genes [19]. Thus, our further investigation was focused on 7q21.3 locus, trying to find out more candidate protooncogenes within this amplified subchromosomal region.

**Table 3:** Amplicons on chromosome 7 detected by digital karyotyping

No. of amplicons	Start Position (bp)	End Position (bp)	Amplification Size (bp)	Known genes contained within amplicons
1	85669352	85675348	5996	--
2	90176250	90233199	56949	PFTK1
3	92170683	92181579	10896	--
4	92402830	92489679	86849	CDK6
5	92525776	92559603	33827	--
6	92621928	92628443	6515	--
7	94153659	94160414	6755	CASD1
8	94187248	94227832	40584	***SGCE***
9	95575453	95593279	17826	***DYNC1I1***
10	95625240	95626171	931	***DYNC1I1***
11	95626365	95634906	8541	***DYNC1I1***
12	95857177	95888724	31547	***SLC25A13***
13	95984342	95992052	7710	--
14	96016428	96091391	74963	--
15	96091675	96098719	7044	--
16	96223512	96436326	212814	SHFM1
17	96566511	96585224	18713	--
18	100008093	100031196	23103	ZCWPW1, MEPCE
19	100188936	100202216	13280	FBXO24, PCOLCE
20	100212010	100322730	110720	MOSPD3, TFR2, ACTL6B, GNB2, GIGYF1, POP7, EPO
21	100514082	100516140	2058	--
22	100594842	100615369	20527	MUC12
23	100619990	100633128	13138	MUC12
24	100899076	100900255	1179	--
25	100920401	100933959	13558	--
26	101043124	101046675	3551	EMID2
27	101047291	101056595	9304	EMID2
28	101542859	101587823	44964	CUX1
29	101708443	101725375	16932	CUX1
30	116320004	116359562	39558	MET
31	116359697	116359984	287	MET
32	116516633	116651559	134926	CAPZA2, ST7, ST7OT1, ST7OT4
33	116832388	116859422	27034	ST7, ST7OT3
34	116986284	117113342	127058	ASZ1
35	117258567	117275904	17337	CFTR
36	117301541	117312368	10827	CFTR
37	117348151	117357569	9418	CTTNBP2
38	117371511	117376667	5156	CTTNBP2
39	117487299	117511084	23785	CTTNBP2
40	117575700	117576160	460	--
41	117604668	117718500	113832	--
42	117727390	117773948	46558	--
43	117862904	117882935	20031	ANKRD7
44	117888893	117908976	20083	--
45	118228295	118274830	46535	--
46	118275399	118309895	34496	--
47	118442613	118473838	31225	--
48	118574464	118640670	66206	--
49	149702946	149746115	43169	--
50	153635222	153636533	1311	DPP6

### Genomic amplification of SGCE, PEG10, DYNC1I1 and SLC25A13 in HCC

**Figure 1:**
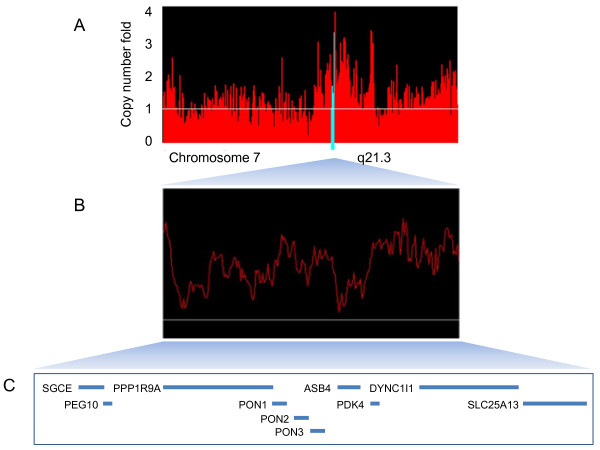
**Schematic map of amplicons at 7q21.3 in HCC**. (A) Digital karyotyping analysis of chromosome 7 in HCC revealed amplicons at 7q21.3 (indicated by blue bars). (B) Zoom in of the 7q21.3 amplicons. (C) RefSeq genes located within the 7q21.3 amplicons.

**Figure 2:**
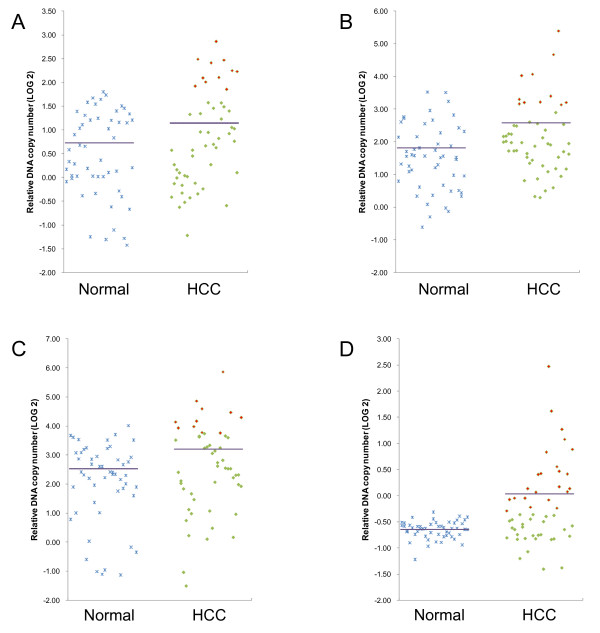
**Real-time quantitative PCR validation of digital karyotyping data**. Totally 52 normal individuals and 52 HCC samples were examined for genomic DNA level of (A) SGCE, (B) PEG10, (C) DYNC1I1 and (D) SLC25A13 respectively by real-time quantitative PCR. The purple horizontal line represented an average of genomic DNA level of each group. HCC samples which gained genomic amplification of examined genes were shown by red squares.

To validate genomic amplifications of SGCE, DYNC1I1 and SLC25A13 detected by digital karyotyping analysis, real-time quantitative PCR was performed in 52 HCC samples and 52 normal individuals. PEG10, a gene located very closely to SGCE in a head-to-head manner, was also selected for examination as a positive control, because both amplification and up-regulation of PEG10 have been observed in HCC [16,23]. The schematic illustration of genes located within subchromosomal region of 7q21.3 (from 94,187,248 bp to 95,968,724 bp) was shown in Figure 1. Amplification of genomic DNA was defined as the DNA level of a HCC sample exceeded the mean plus 2 standard deviations of 52 normal individuals [24]. As shown in Figure 2, genomic amplification of SGCE, PEG10, DYNC1I1 and SLC25A13 was observed in 11 (21%), 11 (21%), 11 (21%) and 23 (44%) of the 52 HCC samples respectively. In the cases of SGCE, PEG10, and SLC25A13 genes, significant differences were observed between HCC samples and normal individuals, with *p *< 0.05 after Bonferroni correction. However, the DYNC1I1 gene was likely to be a nominal one, with *p *value of 0.07 after Bonferroni correction (Additional File 2).

### Up-regulated expression of SGCE, PEG10 and DYNC1I1 in HCC

**Figure 3:**
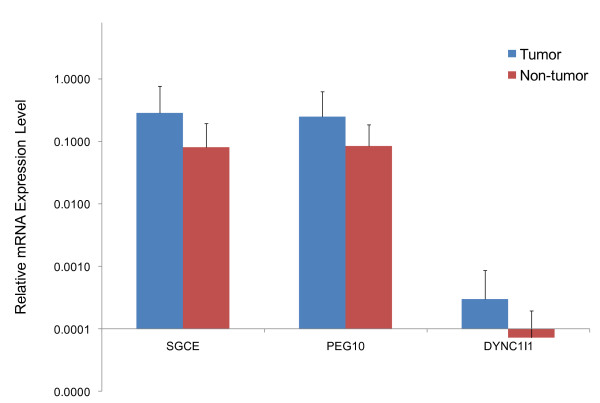
**Up-regulated gene expression of SGCE, PEG10 and DYNC1I1 in HCC**. Real-time quantitative RT-PCR was performed in 32 pairs of HCC and corresponding nontumorous liver tissues. Significant up-regulation of mRNA expression level in HCC was observed in SGCE and PEG10 genes (*p *< 0.05 after Bonferroni correction), and DYNC1I1 was considered as a nominal gene differently expressed in HCC with *p *value of 0.09 after Bonferroni correction. Blue bars represented expression level of HCC tumorous tissues, red bars represented nontumorous liver tissues, and horizontal bars represented SD values.

The transcript expression level of SGCE, PEG10, DYNC1I1 and SLC25A13 genes in tumorous and paired nontumorous tissues were examined in 32 HCC patients by real-time quantitative RT-PCR. As shown in Figure 3 and Additional File 3, mRNA expression level of SGCE, PEG10 and DYNC1I1 genes were up-regulated in tumorous liver tissues compared with corresponding nontumorous counterparts. However, no significant difference was observed in expression level of SLC25A13 between tumorous and nontumorous liver tissues (data not shown). Taken together, these results suggested that SGCE, PEG10 and DYNC1I1 were putative oncogenes at the amplified 7q21.3 locus in HCC.

## Discussion

In the present study, we applied 454 GS FLX, one of the next-generation sequencing systems, in large-scale sequencing of digital karyotyping library of HCC. To the best of our knowledge, no digital karyotyping data of HCC has been published in public. In most of the digital karyotyping libraries of human brain and colon available from public database http://cgap.nci.nih.gov/SAGE/DKViewHome, tags matched to human genome sequences accounted for 62~69% of total genomic tags (Additional File 4). In our sequencing results, 64% (529,162) of the total 821,252 genomic tags were mapped tags, which was quite consistent with published data. Sequentially aligning these tags along each chromosome led to detection of subtle subchromosomal alterations occurred in HCC, among which the amplification of 7q21.3 drew our special attention.

The role of 7q21 amplification either in HCC cell lines or in primary HCC tumors have been implicated in previous studies [16,23,25]. However, within the subchromosome region7q21.3, only PEG10 has been identified as a potential target gene related to progressive development of HCC [16,23]. Our study suggested that another two genes, SGCE and DYNC1I1, may also be HCC-related protooncogenes within the 7q21.3 locus. Genomic amplification of PEG10, SGCE, DYNC1I1 and SLC25A13 were detected by digital karyotyping analysis of HCC, and validated through real-time quantitative PCR. Among 52 HCC samples we examined, eleven (21%) of them showed genomic amplification of SGCE, PEG10 and DYNC1I1 respectively, and 23 (44%) of them exhibited genomic amplification of SLC25A13. Further examinations on mRNA expression level of these genes found that SGCE, PEG10 and DYNC1I1 were significantly up-regulated in HCC tumorous tissues compared with the paired nontumorous counterparts. Taken together, these findings indicated that in addition to PEG10, SGCE and DYNC1I1 were likely to be candidate targets for the 7q21.3 locus in HCC.

PEG10 has been identified as a putative target gene for the amplification at 7q21, as well as a progression related biomarker for HCC [16,23]. SGCE, the epsilon member of sarcoglycan family, was demonstrated to be involved in myoclonus-dystonia syndrome (MDS) which is characterized by rapid myoclonic jerks and dystonia [26]. Recent studies shed light on a role SGCE may play in tumors, as it was up-regulated in a parallel way with PEG10 in both B-cell chronic lymphocytic leukemia and HCC, and showed low or null microsatellite instability in colorectal cancer [19,27,28]. However, since the function of SGCE and the mechanism of how it participate in tumor progression remains unknown up to now, its differential expression in HCC could only be regarded as a potential tumor-related marker without any concrete function.

DYNC1I1 gene encoded dynein intermediate chains 1, a component of cytoplasmic dynein complex, and participated in recognizing, binding and transporting vesicles, proteins, and RNAs *et al *[29,30]. DYNC1I1 was also reported to be a reliable positive marker in testing bone marrow stromal cells prepared for tissue engineering and cell therapy [31]. Our study indicated DYNC1I1 was amplified and up-regulated in HCC, pointing to a possible association between this gene and hepatocarcinogenesis. Further studies on DYNC1I1 as well as SGCE are needed to explain the exact roles they may play in HCC and/or other tumors.

## Conclusions

In this study, we examined chromosomal aberrations of HCC on a genome-wide scale and found that subchromosomal region of 7q21.3 was amplified in HCC. Genomic amplification of PEG10, SGCE, DYNC1I1 and SLC25A13 genes which located within the 7q21.3 locus were detected in HCC, and up-regulated expression of genes was also found in cases of PEG10, SGCE and DYNC1I1. These results suggested that besides PEG10, SGCE and DYNC1I1 may also be probable target genes in the subchromosomal region of 7q21.3. Further studies are needed to explore functional roles of PEG10, SGCE and DYNC1I1 genes might play in the initiation and/or progression of HCC.

## Abbreviations

HCC: hepatocellular carcinoma; PEG10: paternally expressed 10; SGCE: sarcoglycan, epsilon; DYNC1I1: dynein, cytoplasmic 1, intermediate chain 1; SLC25A13: solute carrier family 25, member 13.

## Competing interests

The authors declare that they have no competing interests.

## Authors' contributions

HD designed the experiments, participated in most experiments and drafted the manuscript. HYZ, JPL, HDY and YLK collected the clinical samples. YYC performed the bioinformatics analyses. YS and WRJ constructed the digital karyotyping library and performed qPCR experiments. GPZ and SYW participated in manuscript drafting and revision. All authors read and approved the final manuscript.

## Pre-publication history

The pre-publication history for this paper can be accessed here:

http://www.biomedcentral.com/1755-8794/4/60/prepub

## Supplementary Material

Additional file 1 **Subchromosomal regions of amplification and deletion in HCC detected by digital karyotyping**. Digital karyotyping revealed that subchromosomal amplification (fold change ≥3) and deletion (fold change ≤0.1) occurred in multiple chromosomes in HCC. Click here for file

Additional file 2 **Raw data of real-time quantitative PCR validation for genomic DNA level of SGCE, PEG10, DYNC1I1 and SLC25A13 genes**. Raw data of genomic DNA level of SGCE, PEG10, DYNC1I1 and SLC25A13 genes in 52 normal individuals and 52 HCC samples were presented in this file. Relative genomic DNA level of SGCE, PEG10, DYNC1I1 and SLC25A13 genes were calculated according to the formula 2^Ct(LINE-1)-Ct(target gene)^. Student's *t *test and Bonferroni correction were applied in the statistical analysis of raw data. Click here for file

Additional file 3 **Raw data of real-time quantitative RT-PCR detection for SGCE, PEG10 and DYNC1I1 genes**. Raw data of SGCE, PEG10 and DYNC1I1 gene expression level in 32 pairs of HCC and corresponding nontumorous liver tissues determined by real-time quantitative RT-PCR were presented in this file. Relative expression level of DYNC1I1, SGCE and PEG10 genes were calculated according to the formula 2^Ct(GAPDH)-Ct(target gene)^. Student's *t *test and Bonferroni correction were applied in the statistical analysis of raw data. Click here for file

Additional file 4 **Data summary of 23 digital karyotyping libraries in public database**. In the 23 digital karyotyping libraries available at http://cgap.nci.nih.gov/SAGE/DKViewHome, percentage of tags matched to human genome varied from 48% to 69%, while most of them were between 62% and 69%. Click here for file
