# Supplementary material for: Digital karyotyping reveals probable target genes at 7q21.3 locus in hepatocellular carcinoma
Source: BMC Med Genomics. 2011 Jul 19;4:60. doi: 10.1186/1755-8794-4-60 (PMC3152898; doi:10.1186/1755-8794-4-60)
Supplement: Additional file 1 — Subchromosomal regions of amplification and deletion in HCC detected by digital karyotyping. Digital karyotyping revealed that subchromosomal amplification (fold change ≥3) and deletion (fold change ≤0.1) occurred in multiple chromosomes in HCC. [file 1755-8794-4-60-S1.DOC]

**Subchromosomal regions of amplification in HCC**

| Chromosome | Max Tag Ratio | Amplification Size | Start Position | End Position |
| --- | --- | --- | --- | --- |
| 1 | 3.22 | 5587 | 16904214 | 16909801 |
| 1 | 3.37 | 96159 | 16974706 | 17070865 |
| 1 | 4.23 | 344054 | 143752449 | 144096503 |
| 2 | 3.71 | 3240829 | 91498223 | 94739052 |
| 4 | 3.23 | 3497 | 519704 | 523201 |
| 6 | 8.96 | 167923 | 93453733 | 93621656 |
| 6 | 8.18 | 57284 | 144870285 | 144927569 |
| 6 | 3.06 | 5745 | 155328636 | 155334381 |
| 7 | 3.19 | 5996 | 85669352 | 85675348 |
| 7 | 3.7 | 56949 | 90176250 | 90233199 |
| 7 | 3.17 | 10896 | 92170683 | 92181579 |
| 7 | 3.9 | 86849 | 92402830 | 92489679 |
| 7 | 3.2 | 33827 | 92525776 | 92559603 |
| 7 | 3.21 | 6515 | 92621928 | 92628443 |
| 7 | 3.01 | 6755 | 94153659 | 94160414 |
| 7 | 3.23 | 40584 | 94187248 | 94227832 |
| 7 | 3.18 | 17826 | 95575453 | 95593279 |
| 7 | 3.32 | 931 | 95625240 | 95626171 |
| 7 | 3.24 | 8541 | 95626365 | 95634906 |
| 7 | 3.17 | 31547 | 95857177 | 95888724 |
| 7 | 3.03 | 7710 | 95984342 | 95992052 |
| 7 | 3.85 | 74963 | 96016428 | 96091391 |
| 7 | 3.01 | 7044 | 96091675 | 96098719 |
| 7 | 4.23 | 212814 | 96223512 | 96436326 |
| 7 | 3.23 | 18713 | 96566511 | 96585224 |
| 7 | 3.25 | 23103 | 100008093 | 100031196 |
| 7 | 3.16 | 13280 | 100188936 | 100202216 |
| 7 | 3.77 | 110720 | 100212010 | 100322730 |
| 7 | 3.03 | 2058 | 100514082 | 100516140 |
| 7 | 3.34 | 20527 | 100594842 | 100615369 |
| 7 | 3.34 | 13138 | 100619990 | 100633128 |
| 7 | 3.14 | 1179 | 100899076 | 100900255 |
| 7 | 3.38 | 13558 | 100920401 | 100933959 |
| 7 | 3.21 | 3551 | 101043124 | 101046675 |
| 7 | 3.23 | 9304 | 101047291 | 101056595 |
| 7 | 3.36 | 44964 | 101542859 | 101587823 |
| 7 | 3.25 | 16932 | 101708443 | 101725375 |
| 7 | 3.68 | 39558 | 116320004 | 116359562 |
| 7 | 3.07 | 287 | 116359697 | 116359984 |
| 7 | 4.74 | 134926 | 116516633 | 116651559 |
| 7 | 3.17 | 27034 | 116832388 | 116859422 |
| 7 | 3.84 | 127058 | 116986284 | 117113342 |
| 7 | 3.12 | 17337 | 117258567 | 117275904 |
| 7 | 3.12 | 10827 | 117301541 | 117312368 |
| 7 | 3.21 | 9418 | 117348151 | 117357569 |
| 7 | 3.13 | 5156 | 117371511 | 117376667 |
| 7 | 3.28 | 23785 | 117487299 | 117511084 |
| 7 | 3.27 | 460 | 117575700 | 117576160 |
| 7 | 3.9 | 113832 | 117604668 | 117718500 |
| 7 | 3.67 | 46558 | 117727390 | 117773948 |
| 7 | 3.3 | 20031 | 117862904 | 117882935 |
| 7 | 3.38 | 20083 | 117888893 | 117908976 |
| 7 | 3.41 | 46535 | 118228295 | 118274830 |
| 7 | 3.2 | 34496 | 118275399 | 118309895 |
| 7 | 3.38 | 31225 | 118442613 | 118473838 |
| 7 | 3.37 | 66206 | 118574464 | 118640670 |
| 7 | 3.69 | 43169 | 149702946 | 149746115 |
| 7 | 3.16 | 1311 | 153635222 | 153636533 |
| 8 | 10.77 | 3106493 | 43953766 | 47060259 |
| 8 | 6.38 | 43841 | 86970973 | 87014814 |
| 8 | 3.03 | 1879 | 143234662 | 143236541 |
| 8 | 3.16 | 14843 | 144278356 | 144293199 |
| 8 | 3.36 | 6276 | 145236444 | 145242720 |
| 9 | 5.59 | 255955 | 67868177 | 68124132 |
| 9 | 3.73 | 32801 | 138859387 | 138892188 |
| 10 | 3.97 | 60604 | 127680205 | 127740809 |
| 14 | 3.31 | 334400 | 18193293 | 18527693 |
| 14 | 3.08 | 188053 | 18667449 | 18855502 |
| 15 | 4.91 | 429232 | 18675978 | 19105210 |
| 15 | 3.46 | 7582 | 19187151 | 19194733 |
| 15 | 3.68 | 39093 | 19220863 | 19259956 |
| 15 | 3.68 | 423968 | 19413162 | 19837130 |
| 15 | 3.45 | 33187 | 19888405 | 19921592 |
| 15 | 3.45 | 14577 | 19934149 | 19948726 |
| 16 | 3.46 | 36486 | 22554756 | 22591242 |
| 16 | 5.14 | 93014 | 33829174 | 33922188 |
| 17 | 11.36 | 1632513 | 32103204 | 33735717 |
| 17 | 3.02 | 966 | 70513539 | 70514505 |
| 17 | 3.22 | 12511 | 78524685 | 78537196 |
| 17 | 3 | 3323 | 78537962 | 78541285 |
| 19 | 18.57 | 89909 | 46698811 | 46788720 |
| 21 | 6.25 | 135131 | 13978136 | 14113267 |
| 22 | 3.11 | 313 | 48738683 | 48738996 |
| X | 24.17 | 3185164 | 58495409 | 61680573 |
| Y | 10.51 | 148640 | 11772519 | 11921159 |

**Subchromosomal regions of deletion in HCC**

| Chromosome | Max Tag Ratio | Deletion Size | Start Position | End Position |
| --- | --- | --- | --- | --- |
| 1 | 0 | 40954 | 739503 | 780457 |
| 1 | 0 | 4411 | 40982456 | 40986867 |
| 1 | 0 | 18524 | 153674092 | 153692616 |
| 1 | 0.1 | 27548 | 167486976 | 167514524 |
| 1 | 0.06 | 17028 | 169734288 | 169751316 |
| 1 | 0.05 | 163 | 169842429 | 169842592 |
| 1 | 0.06 | 510 | 169861173 | 169861683 |
| 1 | 0 | 2528 | 170819652 | 170822180 |
| 1 | 0.02 | 4729 | 174448013 | 174452742 |
| 1 | 0.01 | 10732 | 175080335 | 175091067 |
| 1 | 0.04 | 902 | 175322377 | 175323279 |
| 1 | 0.04 | 481 | 177165304 | 177165785 |
| 1 | 0.02 | 14281 | 178027054 | 178041335 |
| 1 | 0 | 6353 | 180499922 | 180506275 |
| 1 | 0.02 | 9751 | 186303508 | 186313259 |
| 1 | 0.05 | 3783 | 192756090 | 192759873 |
| 1 | 0 | 114173 | 193068479 | 193182652 |
| 1 | 0 | 66123 | 193194944 | 193261067 |
| 1 | 0.1 | 10872 | 193476581 | 193487453 |
| 1 | 0.07 | 20540 | 203615712 | 203636252 |
| 1 | 0.02 | 14876 | 241678320 | 241693196 |
| 2 | 0 | 22389 | 138055 | 160444 |
| 2 | 0.04 | 762 | 4362712 | 4363474 |
| 2 | 0 | 8635 | 15098263 | 15106898 |
| 2 | 0.04 | 9741 | 33812281 | 33822022 |
| 2 | 0.02 | 17214 | 41770030 | 41787244 |
| 2 | 0 | 379 | 42912762 | 42913141 |
| 2 | 0 | 5632 | 123443549 | 123449181 |
| 2 | 0.01 | 6051 | 123802697 | 123808748 |
| 2 | 0 | 25833 | 124586458 | 124612291 |
| 2 | 0.08 | 4464 | 124819094 | 124823558 |
| 2 | 0.02 | 32053 | 126066324 | 126098377 |
| 2 | 0 | 13087 | 133107229 | 133120316 |
| 2 | 0 | 7060 | 136213152 | 136220212 |
| 2 | 0 | 19724 | 137662113 | 137681837 |
| 2 | 0 | 208 | 139026326 | 139026534 |
| 2 | 0 | 72521 | 139047375 | 139119896 |
| 2 | 0.01 | 5306 | 139132369 | 139137675 |
| 2 | 0 | 4815 | 141812419 | 141817234 |
| 2 | 0.08 | 5575 | 141875986 | 141881561 |
| 2 | 0.09 | 9303 | 162107824 | 162117127 |
| 2 | 0.1 | 1271 | 162326983 | 162328254 |
| 2 | 0.06 | 13675 | 166560720 | 166574395 |
| 3 | 0 | 19852 | 153123 | 172975 |
| 3 | 0.07 | 5069 | 59404448 | 59409517 |
| 3 | 0 | 18164 | 76426583 | 76444747 |
| 3 | 0.02 | 276 | 111935668 | 111935944 |
| 3 | 0 | 112195 | 164030139 | 164142334 |
| 4 | 0 | 12094 | 684586 | 696680 |
| 4 | 0 | 21763 | 9559191 | 9580954 |
| 4 | 0 | 27674 | 29206250 | 29233924 |
| 4 | 0.09 | 21033 | 42724445 | 42745478 |
| 4 | 0.02 | 358 | 47489303 | 47489661 |
| 4 | 0 | 15730 | 47501181 | 47516911 |
| 4 | 0 | 26145 | 52569320 | 52595465 |
| 4 | 0.02 | 13388 | 62351695 | 62365083 |
| 4 | 0 | 39286 | 70350195 | 70389481 |
| 4 | 0 | 40398 | 82341517 | 82381915 |
| 4 | 0.05 | 2295 | 96615422 | 96617717 |
| 4 | 0.06 | 533 | 96665013 | 96665546 |
| 4 | 0.09 | 12642 | 106550874 | 106563516 |
| 4 | 0.01 | 35711 | 106577790 | 106613501 |
| 4 | 0.08 | 732 | 154927122 | 154927854 |
| 4 | 0 | 41547 | 155830286 | 155871833 |
| 4 | 0.07 | 11055 | 163789229 | 163800284 |
| 4 | 0 | 33927 | 164158638 | 164192565 |
| 4 | 0.07 | 2980 | 164588237 | 164591217 |
| 5 | 0 | 19159 | 21170 | 40329 |
| 5 | 0.06 | 512 | 12819981 | 12820493 |
| 5 | 0.1 | 16463 | 25805589 | 25822052 |
| 5 | 0.1 | 85 | 25836350 | 25836435 |
| 5 | 0 | 20288 | 44590096 | 44610384 |
| 5 | 0.02 | 8933 | 55843389 | 55852322 |
| 5 | 0 | 10669 | 80664596 | 80675265 |
| 5 | 0 | 38960 | 141787111 | 141826071 |
| 5 | 0.1 | 9336 | 143290587 | 143299923 |
| 5 | 0.06 | 27319 | 144053283 | 144080602 |
| 5 | 0 | 1160 | 144226754 | 144227914 |
| 5 | 0.07 | 2875 | 145650011 | 145652886 |
| 5 | 0.02 | 4153 | 146041165 | 146045318 |
| 5 | 0.1 | 1776 | 146049566 | 146051342 |
| 5 | 0.02 | 16407 | 150258881 | 150275288 |
| 5 | 0.1 | 11014 | 150280396 | 150291410 |
| 5 | 0.1 | 191 | 152507846 | 152508037 |
| 5 | 0 | 1588 | 152766700 | 152768288 |
| 5 | 0 | 6224 | 153988938 | 153995162 |
| 5 | 0.1 | 3520 | 172199677 | 172203197 |
| 5 | 0.09 | 117 | 172354283 | 172354400 |
| 5 | 0 | 3447 | 174634774 | 174638221 |
| 5 | 0 | 0 | 180661399 | 180661399 |
| 6 | 0 | 7795 | 395436 | 403231 |
| 6 | 0.06 | 4741 | 16644793 | 16649534 |
| 7 | 0 | 12231 | 201296 | 213527 |
| 7 | 0.05 | 31142 | 7526566 | 7557708 |
| 7 | 0.02 | 100582 | 12176266 | 12276848 |
| 7 | 0.02 | 3474 | 24713858 | 24717332 |
| 7 | 0.06 | 1620 | 46491891 | 46493511 |
| 7 | 0.08 | 2326 | 54058401 | 54060727 |
| 7 | 0 | 20595 | 54289610 | 54310205 |
| 7 | 0.07 | 451 | 55192623 | 55193074 |
| 7 | 0 | 40632 | 57207445 | 57248077 |
| 7 | 0.01 | 8515 | 77418121 | 77426636 |
| 7 | 0.01 | 275 | 77536198 | 77536473 |
| 7 | 0 | 9621 | 77656816 | 77666437 |
| 7 | 0 | 124165 | 78243633 | 78367798 |
| 8 | 0 | 3534 | 222185 | 225719 |
| 8 | 0.06 | 18006 | 17933701 | 17951707 |
| 8 | 0.06 | 17963 | 17960660 | 17978623 |
| 8 | 0 | 4064 | 24696880 | 24700944 |
| 9 | 0.04 | 282 | 25510788 | 25511070 |
| 9 | 0 | 43417 | 43570699 | 43614116 |
| 9 | 0 | 61571 | 140164689 | 140226260 |
| 10 | 0 | 21133 | 218276 | 239409 |
| 10 | 0.08 | 3003 | 7970465 | 7973468 |
| 10 | 0.08 | 154 | 7986702 | 7986856 |
| 10 | 0 | 46389 | 70049359 | 70095748 |
| 11 | 0.02 | 19080 | 9718391 | 9737471 |
| 11 | 0 | 13479 | 11669902 | 11683381 |
| 11 | 0 | 40564 | 13723220 | 13763784 |
| 11 | 0.01 | 48079 | 16166837 | 16214916 |
| 11 | 0.09 | 28626 | 16228208 | 16256834 |
| 11 | 0 | 25932 | 55152921 | 55178853 |
| 11 | 0.08 | 12831 | 55205085 | 55217916 |
| 11 | 0 | 29644 | 96639486 | 96669130 |
| 11 | 0.01 | 21934 | 98901862 | 98923796 |
| 11 | 0.01 | 60504 | 101329100 | 101389604 |
| 11 | 0.06 | 17524 | 105283715 | 105301239 |
| 11 | 0.04 | 3412 | 105957828 | 105961240 |
| 11 | 0 | 2598 | 108472869 | 108475467 |
| 11 | 0.08 | 7079 | 118224978 | 118232057 |
| 11 | 0 | 10556 | 134419895 | 134430451 |
| 12 | 0 | 9347 | 247129 | 256476 |
| 12 | 0.06 | 404 | 18094811 | 18095215 |
| 12 | 0.02 | 666 | 20725190 | 20725856 |
| 12 | 0.08 | 7213 | 23401074 | 23408287 |
| 12 | 0 | 72198 | 29400054 | 29472252 |
| 12 | 0.04 | 20634 | 33545643 | 33566277 |
| 12 | 0.09 | 15905 | 37501993 | 37517898 |
| 12 | 0 | 21220 | 54072974 | 54094194 |
| 12 | 0 | 865 | 78873786 | 78874651 |
| 12 | 0.08 | 1595 | 104352468 | 104354063 |
| 13 | 0 | 15264 | 19271014 | 19286278 |
| 14 | 0.1 | 26505 | 39246165 | 39272670 |
| 14 | 0.08 | 8399 | 56437338 | 56445737 |
| 14 | 0.03 | 25356 | 62154827 | 62180183 |
| 14 | 0.03 | 961 | 62210878 | 62211839 |
| 14 | 0.03 | 1833 | 62259668 | 62261501 |
| 14 | 0 | 31904 | 63202094 | 63233998 |
| 14 | 0.08 | 27132 | 63246232 | 63273364 |
| 14 | 0.03 | 4093 | 65695019 | 65699112 |
| 14 | 0 | 27493 | 65804737 | 65832230 |
| 14 | 0.01 | 34741 | 70880088 | 70914829 |
| 14 | 0.07 | 2511 | 72270162 | 72272673 |
| 14 | 0.01 | 5729 | 80933745 | 80939474 |
| 14 | 0 | 37035 | 82367583 | 82404618 |
| 14 | 0 | 26592 | 89531828 | 89558420 |
| 14 | 0 | 846 | 89581252 | 89582098 |
| 14 | 0 | 29920 | 91182403 | 91212323 |
| 14 | 0 | 11432 | 106346773 | 106358205 |
| 15 | 0 | 48218 | 20017784 | 20066002 |
| 15 | 0 | 4111 | 100327622 | 100331733 |
| 16 | 0.07 | 76810 | 16517792 | 16594602 |
| 16 | 0 | 431 | 17108869 | 17109300 |
| 16 | 0 | 159794 | 18295118 | 18454912 |
| 16 | 0 | 52597 | 18618703 | 18671300 |
| 16 | 0 | 17142 | 22846392 | 22863534 |
| 16 | 0 | 21825 | 26198122 | 26219947 |
| 16 | 0.06 | 68059 | 33560611 | 33628670 |
| 16 | 0 | 30411 | 76499079 | 76529490 |
| 16 | 0 | 4316 | 88630227 | 88634543 |
| 17 | 0 | 25273 | 84776 | 110049 |
| 18 | 0 | 14112 | 76097897 | 76112009 |
| 19 | 0 | 24014 | 63722161 | 63746175 |
| 20 | 0.05 | 5295 | 14227550 | 14232845 |
| 20 | 0.01 | 19206 | 17482707 | 17501913 |
| 20 | 0 | 28108 | 18485844 | 18513952 |
| 20 | 0 | 53110 | 62379100 | 62432210 |
| 21 | 0 | 52950 | 10590493 | 10643443 |
| 22 | 0 | 138722 | 16886851 | 17025573 |
| X | 0 | 87628 | 3999039 | 4086667 |
| X | 0.01 | 9346 | 5102933 | 5112279 |
| X | 0.09 | 34035 | 5365158 | 5399193 |
| X | 0.09 | 4668 | 6801122 | 6805790 |
| X | 0 | 12810 | 8092679 | 8105489 |
| X | 0.1 | 1606 | 11406723 | 11408329 |
| X | 0.1 | 6712 | 12492514 | 12499226 |
| X | 0.07 | 50882 | 14285743 | 14336625 |
| X | 0 | 31346 | 14597758 | 14629104 |
| X | 0.08 | 18807 | 27190856 | 27209663 |
| X | 0.1 | 37589 | 34669076 | 34706665 |
| X | 0.1 | 2181 | 34773646 | 34775827 |
| X | 0.08 | 8309 | 37904750 | 37913059 |
| X | 0.02 | 30816 | 41780682 | 41811498 |
| X | 0.02 | 58308 | 42163189 | 42221497 |
| X | 0.04 | 415 | 45972621 | 45973036 |
| X | 0.1 | 2569 | 52597180 | 52599749 |
| X | 0 | 21208 | 56949546 | 56970754 |
| X | 0.03 | 191 | 63692002 | 63692193 |
| X | 0.1 | 19488 | 65201008 | 65220496 |
| X | 0.07 | 25148 | 67185935 | 67211083 |
| X | 0.08 | 765 | 68496715 | 68497480 |
| X | 0.02 | 91167 | 69787463 | 69878630 |
| X | 0.07 | 845 | 82269145 | 82269990 |
| X | 0.07 | 76107 | 89730697 | 89806804 |
| X | 0.02 | 26007 | 100667311 | 100693318 |
| X | 0.07 | 1861 | 104952125 | 104953986 |
| X | 0.09 | 20187 | 106072940 | 106093127 |
| X | 0.01 | 32747 | 106280931 | 106313678 |
| X | 0.09 | 16839 | 106459066 | 106475905 |
| X | 0.01 | 4231 | 106501292 | 106505523 |
| X | 0.08 | 13292 | 106521091 | 106534383 |
| X | 0.09 | 2269 | 107041926 | 107044195 |
| X | 0 | 46593 | 108298485 | 108345078 |
| X | 0.08 | 23070 | 109156523 | 109179593 |
| X | 0 | 24528 | 110886334 | 110910862 |
| X | 0 | 41167 | 111644254 | 111685421 |
| X | 0.08 | 19855 | 111697927 | 111717782 |
| X | 0.08 | 30922 | 112274551 | 112305473 |
| X | 0.08 | 6254 | 112406978 | 112413232 |
| X | 0.08 | 8155 | 112697253 | 112705408 |
| X | 0 | 25052 | 113890681 | 113915733 |
| X | 0.08 | 35604 | 113933411 | 113969015 |
| X | 0 | 66867 | 114371463 | 114438330 |
| X | 0.08 | 83958 | 114689490 | 114773448 |
| X | 0 | 11902 | 114971973 | 114983875 |
| X | 0.07 | 397 | 115548360 | 115548757 |
| X | 0.06 | 19869 | 115863629 | 115883498 |
| X | 0.06 | 15903 | 116107092 | 116122995 |
| X | 0.02 | 111834 | 117013956 | 117125790 |
| X | 0.1 | 7650 | 117161477 | 117169127 |
| X | 0 | 66649 | 119965635 | 120032284 |
| X | 0.08 | 336 | 120544599 | 120544935 |
| X | 0.01 | 37749 | 120991738 | 121029487 |
| X | 0.08 | 10313 | 121261997 | 121272310 |
| X | 0.07 | 26686 | 122136980 | 122163666 |
| X | 0 | 40481 | 122686469 | 122726950 |
| X | 0.08 | 35868 | 122920619 | 122956487 |
| X | 0 | 21214 | 122970357 | 122991571 |
| X | 0.07 | 2610 | 123184941 | 123187551 |
| X | 0.07 | 1329 | 123202409 | 123203738 |
| X | 0 | 6767 | 123262920 | 123269687 |
| X | 0 | 10019 | 123725078 | 123735097 |
| X | 0 | 39754 | 124381661 | 124421415 |
| X | 0.05 | 25718 | 125956070 | 125981788 |
| X | 0 | 26899 | 126430194 | 126457093 |
| X | 0.01 | 2199 | 128085189 | 128087388 |
| X | 0.01 | 100449 | 128233322 | 128333771 |
| X | 0.02 | 5165 | 129951424 | 129956589 |
| X | 0 | 21941 | 133185673 | 133207614 |
| X | 0.08 | 1679 | 133805234 | 133806913 |
| X | 0.03 | 16527 | 139345521 | 139362048 |
| X | 0.1 | 40 | 146215959 | 146215999 |
| X | 0 | 11264 | 154494105 | 154505369 |
| Y | 0 | 29174 | 2710947 | 2740121 |
| Y | 0.06 | 15356 | 8130909 | 8146265 |
| Y | 0.08 | 28689 | 9123848 | 9152537 |
| Y | 0 | 45198 | 27128717 | 27173915 |
