# Supplementary material for: Digital karyotyping reveals probable target genes at 7q21.3 locus in hepatocellular carcinoma
Source: BMC Med Genomics. 2011 Jul 19;4:60. doi: 10.1186/1755-8794-4-60 (PMC3152898; doi:10.1186/1755-8794-4-60)
Supplement: Additional file 4 — Data summary of 23 digital karyotyping libraries in public database. In the 23 digital karyotyping libraries available at http://cgap.nci.nih.gov/SAGE/DKViewHome, percentage of tags matched to human genome varied from 48% to 69%, while most of them were between 62% and 69%. [file 1755-8794-4-60-S4.DOC]

| **Library** | **Total tags** | **Mapped tags**  **(percentage)** |
| --- | --- | --- |
| DK_Colon_carcinoma_CL_Co93 | 235282 | 113882 (48%) |
| DK_Brain_Medulloblastoma_CL_H556 | 159686 | 82867 (52%) |
| DK_Brain_Medulloblastoma_CL_H487 | 180585 | 111113 (62%) |
| DK_Brain_Medulloblastoma_CL_MHH1 | 168561 | 103753 (62%) |
| DK_Brain_Medulloblastoma_CL_H721 | 234438 | 144567 (62%) |
| DK_Colon_metastasis_AP_MJC3 | 135021 | 84008 (62%) |
| DK_Brain_Medulloblastoma_CL_H283 | 187755 | 118226 (63%) |
| DK_Brain_Glioblastoma_CL_H693 | 209885 | 132547 (63%) |
| DK_Brain_Glioblastoma_CL_H80 | 160155 | 102022 (64%) |
| DK_Brain_Glioblastoma_CL_H423 | 169257 | 108070 (64%) |
| DK_Brain_Glioblastoma_CL_H542 | 172687 | 111601 (65%) |
| DK_Brain_Glioblastoma_B_H1110 | 194089 | 125532 (65%) |
| DK_Brain_Glioblastoma_CL_H259 | 198357 | 129043 (65%) |
| DK_Brain_Glioblastoma_CL_H336 | 180175 | 119512 (66%) |
| DK_Brain_Glioblastoma_CL_H502 | 148484 | 99180 (67%) |
| DK_Brain_Glioblastoma_CL_H270 | 222210 | 148860 (67%) |
| DK_Colon_metastasis_AP_M10-23 | 182155 | 122179 (67%) |
| DK_Colon_metastasis_AP_MJC1 | 210639 | 142461 (68%) |
| DK_Colon_metastasis_AP_M12-02 | 210414 | 142962 (68%) |
| DK_Colon_carcinoma_CL_Co84 | 186065 | 127512 (69%) |
| DK_Colon_carcinoma_CL_Co90 | 206632 | 143371 (69%) |
| DK_Colon_metastasis_AP_M11-01 | 171621 | 119186 (69%) |
| DK_Colon_metastasis_AP_M12-05 | 90641 | 65079 (72%) |
